# Hardness and Corrosion Behavior of CrMnFeCoNi Alloy Fabricated by Ball Milling and Spark Plasma Sintering

**DOI:** 10.3390/ma17194793

**Published:** 2024-09-29

**Authors:** Rongguang Wang, Sohei Kamada

**Affiliations:** 1 Department of Mechanical Systems Engineering, Hiroshima Institute of Technology, Hiroshima 731-5193, Japan; 2 Graduate School of Science and Technology, Hiroshima Institute of Technology, Hiroshima 731-5193, Japan

**Keywords:** CrMnFeCoNi, high entropy, milling, sinter, hardness, corrosion

## Abstract

The mechanical properties and electrochemical stability of high-entropy alloys are substantially affected by their composition distribution and crystal structure. However, the details concerning the conditions of milling and sintering for sintered alloys have rarely been reported. In this work, a series of CrMnFeCoNi alloys were fabricated by ball milling and spark plasm sintering for different periods. Their crystal structure, density, hardness, and corrosion resistance were investigated. As a result, a partial alloying of Cr, Mn, Fe, Co, and Ni was achieved by ball milling. However, Cr-rich particles, including Mn, were formed in the milled powders. The sintered alloys inherited the Cr-rich particles to form Cr-rich zones. The formation and change of chromium carbide were also confirmed in sintered alloys. Extended milling or sintering to 12 h achieved high hardness and corrosion resistance for the sintered alloys. The Cr-rich zones showed high hardness and Kelvin potential, which affect both the hardness and the corrosion resistance.

## 1. Introduction

CrMnFeCoNi (Cantor alloy) [1,2] contains equimolar metal elements of Cr, Mn, Fe, Co, and Ni with a high entropy. It exhibits various excellent properties over a wide temperature range [3,4,5,6,7]. Apart from the high ultimate tensile strength of 1280 MPa (77 K)~759 MPa (293 K) [3], its other properties include the high toughness of 219 MPa·m^1/2^ at 77 K [3], high ductility at elongation > 0.6 [3,4], noble pitting corrosion potential of 460 mV vs. SSE in a NaCl solution [8] at room temperature, and high critical resolved shear stress of nearly 24 MPa at 1280 K [6,7]. Therefore, it attracts much interest from researchers and engineers as a potential structural material.

CrMnFeCoNi alloy can be fabricated by either casting (melting and solidification) [1,2,4,9], sintering (powder metallurgy) [5,10,11,12], or other methods [8,13,14,15,16]. Compared with the more frequently studied casting, sintering has a great economic advantage in saving materials and energy [17]. On the other hand, the element distribution in sintered alloys is usually more inhomogeneous than that of casted ones. One example is the Cr, Mn, and O enrichment in spark-plasma-sintered alloys [10]. The zones with specific element enrichment generally affect the mechanical properties and electrochemical stability. In addition, the impurities of carbon or oxygen from raw powders also play essential roles in the above properties. Therefore, the relationship between the specific microstructure, the mechanical properties, and the electrochemical stability of sintered alloys needs to be sufficiently clarified. Until now, many CrMnFeCoNi alloys have been sintered under specific conditions, and their mechanical properties have been studied [5,10,11,12,18,19,20]. However, the detailed formation behavior of element inhomogeneity in milling and sintering has not been systemically investigated, and its influence on the above properties has not been clarified.

Moreover, the high corrosion resistance of high-entropy alloys has frequently been demonstrated [8,15,16]. However, the corrosion resistance of CrMnFeCoNi alloy is still not comparable with conventional stainless steels in a NaCl solution [9,21,22,23], even though significant amounts of Cr, Co, and Ni are contained within it. The corrosion resistance of this type of alloy largely depends on the fabrication methods and conditions used [9,13,20,22,24,25,26], particularly low for the sintered ones [27]. Until now, corrosion studies on sintered CrMnFeCoNi have been lacking. It is urgent to investigate how the inhomogeneous element distribution affects the corrosion resistance of this alloy. Knowing the relationship between corrosion behavior and Cr-rich zones is especially important to broaden its field applications.

Based on the above knowledge, this work investigated the influence of fabrication conditions on a series of spark-plasma-sintered alloys, including ball milling and sintering times. The mechanical properties, such as hardness and corrosion resistance, and the electrochemical stability were investigated and discussed with regard to the analyzed microstructure.

## 2. Experimental Details

### 2.1. Ball Milling and Sintering

Metal powders of chromium (Cr; mean diameter: 10 μm; purity: 98%), manganese (Mn; 10 μm; 99.9%), iron (Fe; 3~5 μm; 99.9%), cobalt (Co; 5 μm; 99%), and nickel (Ni; 3~5 μm; -) were purchased from Kojundo Chemical Laboratory Co. Ltd. Note that the purity does not consider any non-metallic element. An equimolar power with a total weight of 10 g was milled in a mixing bowl (Fe-Cr alloy; 45mL), which was safely locked to a planetary mono mill machine (PULVERISETTE 6 classic line, FRITSCH Yokohama, Japan). The milling was conducted at 300 rpm for total times of 0.7 h, 6.0 h, or 12.0 h. Overheating was avoided by a 1.0 h running followed by a 0.5 h pause. High-hardness stainless steel balls (Fe-Cr alloy; φ3.5 mm, 90 g in total; ball-to-powder mass ratio: 9) were ultrasonically cleaned in pure water (0.6 ks × 2 times) and acetone (0.6 ks × 2 times) before use in the mixing bowl. The bowl with the balls and powders was vacuumed using an over pot with a pump ((operation: 0.6 ks, pause: 0.3 ks) × 3 cycles) and finally filled with argon gas. Lubricants were not used in the milling to avoid foreign composition. Some Fe, Cr, and C atoms in the balls might have been transferred to the powder. The mixed powers were referred to according to their milling time as BM0.7h, BM6h, and BM12h.

The ball-milled powder was put into a φ10 mm graphite mold and then sintered at 1073 K under a vacuum (<10 Pa) in a spark plasma sintering (SPS) machine (plasma kit, CSP-KIT-02121, S.S. Alloy Co., Ltd., Higashi Hiroshima, Japan) under a vertical pressure of 32.7 MPa. Hereinafter, the sintered specimens are referred to by their milling time and sintering time as BM0.7S0.5, BM0.7S6, BM0.7S12, B6S0.5, B6S6, B6S12, B12S0.5, B12S6, and B12S12. The specimens were finally polished with #2000 emery paper, ultrasonically cleaned with water and acetone, and dried with a hot dryer before the following tests.

### 2.2. Analysis of Density, Crystal Structure, and Composition Distribution

The density of the sintered specimens was determined by the Archimedes method in water using wax to enclose the opening holes. The crystal structures of both powers and sintered specimens were measured by an X-ray diffraction apparatus (Ultima IV, Rigaku), using Cu Kα X-rays (wavelength: 0.15418 nm) generated at 40 kV and 40 mA, at a scanning rate of 3 °/min and a sampling step of 0.02°. The chemical compositions of the powders and sintered specimens were analyzed using an energy-dispersive X-ray spectroscope (SEM-EDS; JED-2300, JEOL Ltd., Tokyo, Japan) attached to a scanning electron microscope (SEM; JOEL-6510A, JEOL Ltd.).

### 2.3. Measure of Hardness and Corrosion Resistance

The deformation resistance of the sintered specimens was measured on nine sites at a load of 9.8 N with a holding time of 12.5 s using a Vickers hardness tester (AVK-CO, Akashi Co. Ltd., Akashi, Japan). In addition, nanoindentation was used in microzones to measure hardness. For corrosion resistance, a 5 mm^2^ exposed area of the upper side of the specimen was used by sealing other surfaces with silicone sealant. A calomel electrode (Hg/Hg_2_Cl_2_; SCE) was used as the reference electrode, and a platinum plate was used as the counter electrode for the polarization measurement. A 3.5% NaCl aqueous solution at 303 ± 1 K was used for the polarization. The solution was deaerated with nitrogen gas for 0.5 h before and during the test. The potential was firstly kept at −1.0 V (vs. SCE) for 120 s, followed by holding at the natural potential for 600 s. The potential was then swept from −0.4 to 0.4 V (vs. OCP) at a scanning rate of 0.333 mV/s. A confocal laser microscope (LEXT OLS-4000, OLYMPUS, Tokyo, Japan) was used for surface observations before and after polarization. In addition, the SKFM method was applied to obtain the potential difference at the nanometer level.

## 3. Results and Discussion

### 3.1. Morphology, Composition, and Crystal Structure of Raw and Mixed Powders

**Figure 1:**
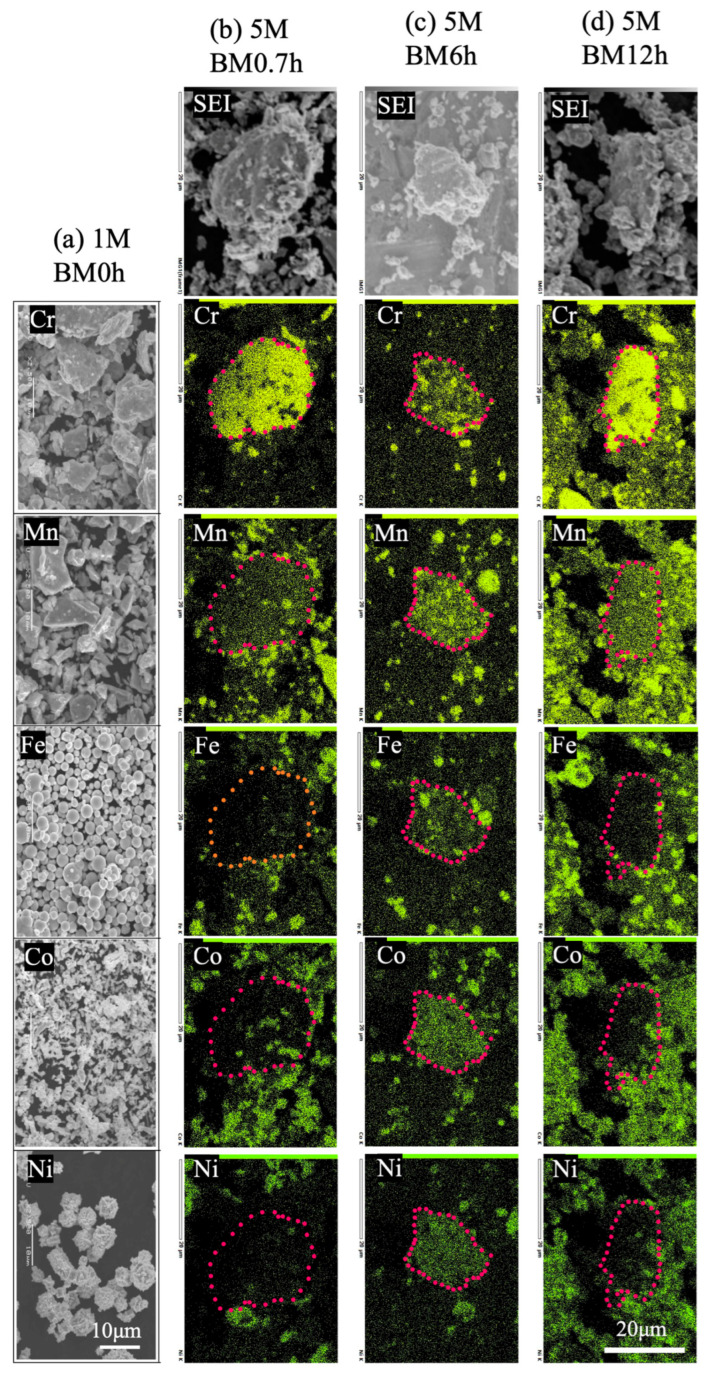
(**a**) Raw powders of Cr, Mn, Fe, Co, and Ni. Element mappings of CrMnFeCoNi powders after (**b**) 0.7 h, (**c**) 6.0 h, and (**d**) 12.0 h of milling.

Figure 1a shows the SEM images of the raw powders of Cr, Mn, Fe, Co, and Ni. Their sizes correspond well to the data specified by the maker. The top images of Figure 1b–d show the mixed powders after milling for 0.7, 6.0, and 12.0 h. Particles larger and smaller than the raw ones, ~30 μm for BM0.7 and ~20 μm for BM6/BM12, can be seen. From the element mapping of the mixed powders, locally aggregated specific elements can be observed from some larger particles at the micrometer level. From the BM0.7h powder, large particles enriched with Cr were detected, which also contained evenly dispersed Mn. Much less Fe, Co, and Ni can be found there. Meanwhile, uncrushed Mn, Fe, Co, and Ni particles can also be detected. In BM6h powder, well-dispersed Cr and Mn were included in some large particles, with a higher Fe, Co, and Ni content ratio than BM0.7h. The uncrushed Mn, Fe, Co, and Ni particles decreased. This result indicates that the milling of all elements proceeded and that there was an explicit affinity between Cr and Mn. The result from BM12h shows more dispersive aspects than others. However, in some Cr-rich particles, the high affinity between Cr and Mn is apparent, whereas the high Fe, Co, and Ni content cannot be easily included. According to Figure 1, we can determine the proceeding mixing of raw powders by milling; however, blending cannot reach the atomic level. BM12h commonly has a high level of mixing; however, the aggregation of Cr and Mn is still remarkable. Such aggregation perhaps can be attributed to the high affinity among Cr, Mn, O, and C. Oxygen (O) and carbon (C) were confirmed from the raw powders and the Cr-rich particles by EDS analysis. The thermal stability of the oxides of Mn and Cr was confirmed to be higher than that of other metals [28]. Benjamin classified the powder change in mechanical alloying as the sequence of (1) particle flattening, (2) lamination bonding of flat particles, (3) formation of equiaxial particles, (4) random orientation junction of particles, and (5) steady state [29]. Although the results of this study are not completely confident, BM0.7h is considered to be in the first stage, BM6h in the second stage, and BM12h in the middle of the fourth and fifth stages.

**Figure 2:**
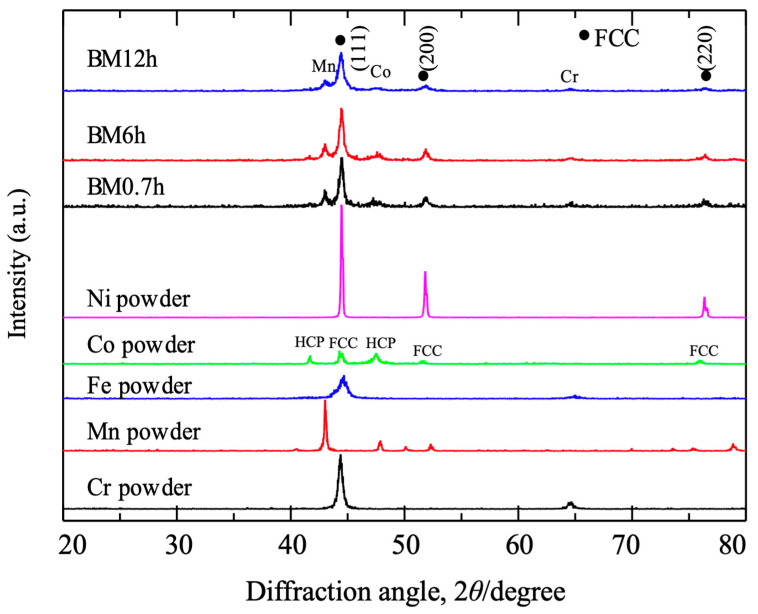
X-ray diffraction patterns of raw and mixed powders milled for different periods.

Figure 2 shows the XRD patterns of the raw and the mixed powders. The raw powders show a body-centered cubic (bcc) structure for Cr and Fe, a complex cubic structure for Mn, a mixed face-centered cubic (fcc), a hexagonal closed packed (hcp) structure for Co, and an fcc structure for Ni. The prominent diffraction peaks from the mixed powders are almost identical to those of the Ni powder (fcc). Several weak peaks originated from Mn, Co, and Cr powders. This result is evident in the alloying among these elements in milling, so-called mechanical alloying (MA), with the substitution of the nickel atoms in the fcc lattice of nickel with other atoms. On the other hand, a few elements remained unchanged in their original crystal structure, which produced the corresponding weak peaks. The diffraction peaks from the mixed powders are wider than the raw Ni powder, especially after 12.0 h milling. This also indicates the distortion of the nickel lattice with the substitution of other elements. In BM12h, except for the broadened peak of Mn, the peaks from the raw metals are much weaker than in BM0.7h and BM6h, indicating the further proceeding of alloying. Ji reported an increased lattice constant from 0.3519 to 0.3536 nm and a decreased crystalline size from 21.1 to 12.7 nm by extending the same alloy’s milling time from 12 to 60 h [12]. As a comparison, the lattice constant and crystalline size corresponding to Figure 2 are shown in Table 1. As with [12], the lattice constant increased with the milling time, even though the time was shorter than 12 h. Perhaps also due to the short milling time, the decrease in crystalline size was not apparent when extending the milling.

### 3.2. Composition and Crystal Structure of Sintered Materials

**Figure 3:**
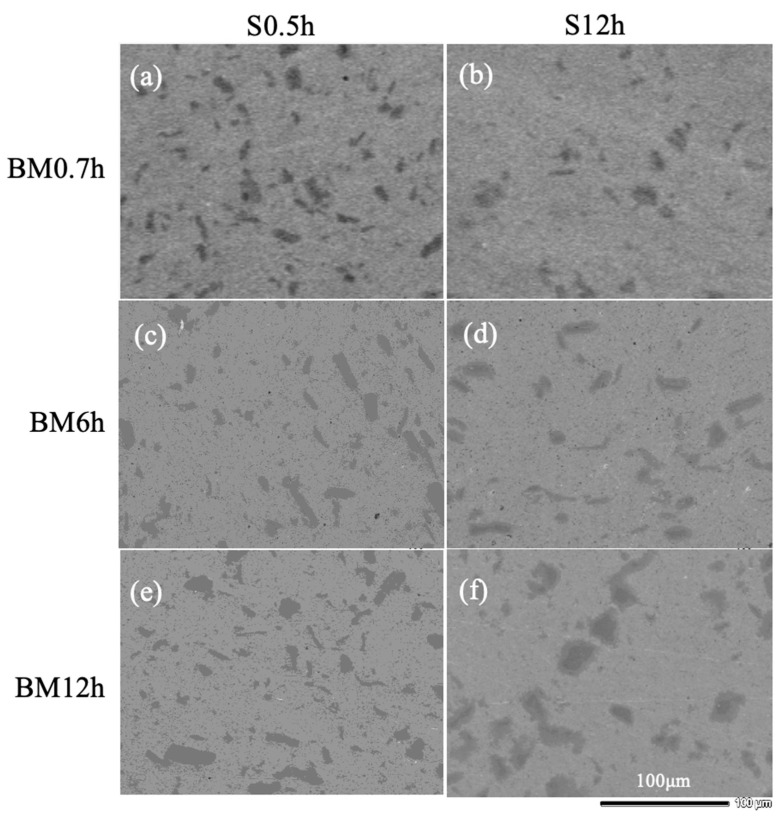
SEM images of sintered CrMnFeCoNi alloys with different periods of milling and sintering. (**a**) B0.7S0.5, (**b**) B0.7S12, (**c**) B6S0.5, (**d**) B6S12, (**e**) B12S0.5, and (**f**) B12S12.

Figure 3 shows the SEM images of several sintered specimens fabricated with different milling and sintering times. For each case, black/grey zones with a length/width of ~20 μm, distinct from the matrix, were found. Their size did not change with the increase in the milling time. An obvious contrast for the S12h seemed to be separated for specimens after 6.0 or 12.0 h milling, indicating a probable diffusion.

**Figure 4:**
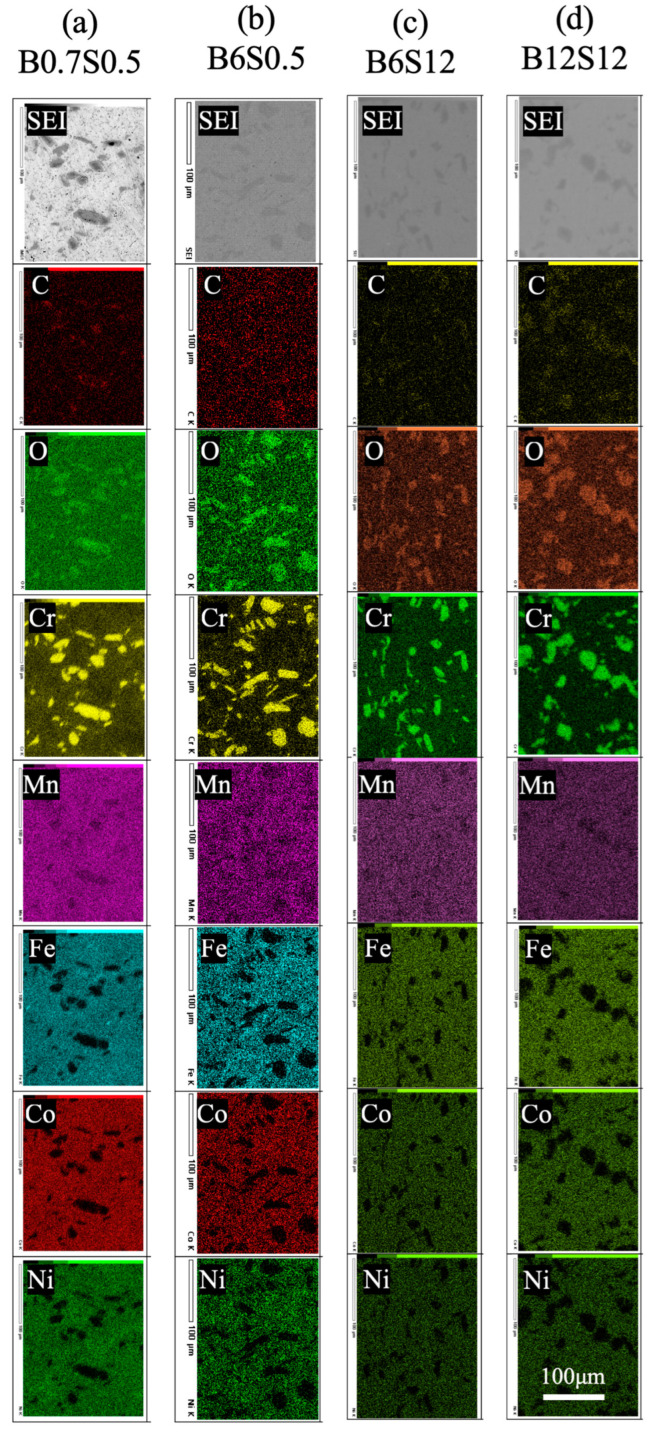
Element mapping of sintered CrMnFeCoNi alloys with different periods of milling and sintering. (**a**) B0.7S0.5, (**b**) B6S0.5, (**c**) B6S12, and (**d**) B12S12.

Figure 4 shows the elemental mapping of the sintered B0.7S0.5, B6S0.5, B6S12, and B12S12, analyzed by SEM-EDS. The matrix shows a homogeneous Cr, Mn, Fe, Co, and Ni distribution. In contrast, the black/grey zones show concentrated Cr, Mn, C, and O. The affinity between Cr and Mn remained after sintering, almost the same as in the powder analysis (Figure 2). C and O should originate from the raw powders detected from the EDS analysis: 3.9~19.2 atom% carbon and 1.0~5.6 atom% oxygen. Generally, C and O have a high affinity for Cr and Mn [28]. The Cr-rich zones were inherited from milling, and were difficult to dissolve in sintering. This might also relate to the slow diffusion rate in the high-entropy alloy [2].

**Figure 5:**
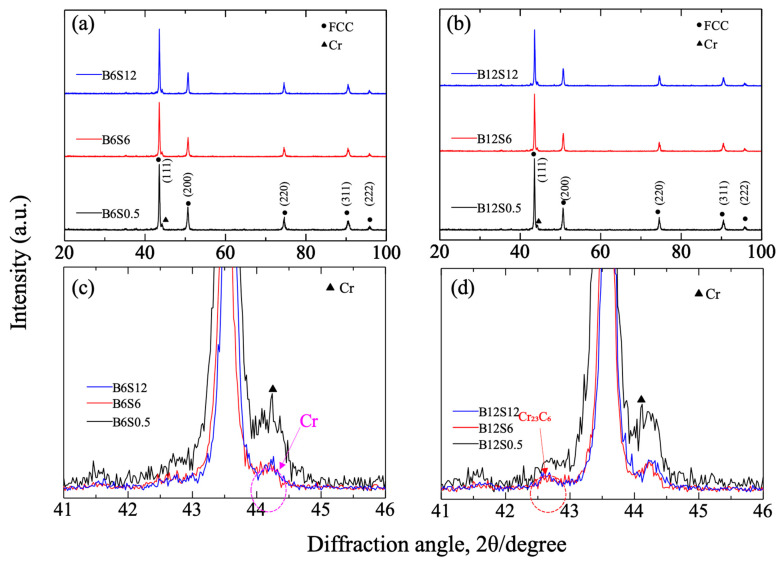
X-ray diffraction patterns of sintered CrMnFeCoNi alloys with different periods of milling and sintering. (**a**) alloys with 6.0 h milling, (**b**) alloys with 12.0 h milling, (**c**) enlarged spectrum for alloys with 6.0 h milling, and (**d**) enlarged spectrum for alloys with 12.0 h milling.

Figure 5 shows the XRD pattern of sintered specimens with 6.0 and 12.0 h milling. The pattern of the 0.7 h milled ones is almost the same as Figure 5. In either case, nearly the same peaks corresponding to an fcc structure were confirmed. All diffraction peaks were sharp. From the enlarged spectrum (Figure 5c,d), a Cr peak was confirmed at 44.2°, with a maximum intensity at 0.5 h sintering and decreasing at 6.0 and 12.0 h. This result suggests the dissolution of the enriched Cr into the matrix in sintering. However, 12.0 h sintering cannot further promote this dissolution. In the case of 12.0 h milling, a small peak corresponding to Cr_23_C_6_ was found near 42.6°, whose intensity also became minor from 0.5 to 6.0/12.0 h sintering periods, showing the decomposition of the carbide but with a limit from 6.0 to 12.0 h sintering. Its amount was independent of milling time. As mentioned before, the carbon atoms originated from the impurity of powders. Accordingly, except for the solid-solutioned Mn, C, and O atoms in the Cr-rich zone, the Cr_23_C_6_ crystals were also contained.

The lattice constants and crystalline sizes were calculated from the diffraction angles and their widths from the plane (200). No significant change trend can be found among the sintered alloys. However, the lattice constants of 3.597~3.609 nm are larger than the milled powders (0.3525~0.3532 nm), indicating the progress of sintering. The increased crystalline size from 22~18 nm (milled powders) to 26~30 nm (sintered alloys) corresponds to the sintering progress.

### 3.3. Density and Hardness of Sintered Materials

**Figure 6:**
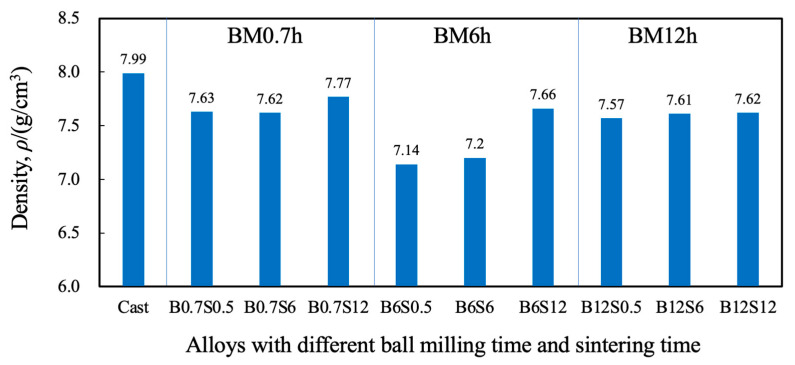
Density of sintered CrMnFeCoNi alloys with different periods of milling and sintering.

Figure 6 shows the density measured by the Archimedes method. Compared to the value of a casted alloy (7.987 g/cm^3^) [18], the sintered ones show relatively small values due to the porosity formed during sintering. The density tended to increase with sintering time, indicating a decrease in porosity. Note that error bars are not shown because of limited measurements. The sintering progress can be clarified as (1) the initial stage of spheroidization: the neck formation/growth and formation of the closed pore; (2) the middle stage: the Oswald growth of the pore and diffusion of vacancies to the particle’s surface, resulting in densification; and (3) the final stage: the isolation of closed pores and their diffusion outside, resulting in the disappearance of pores [30]. According to Figure 6, 0.5 h sintering should be in the middle stage and 12.0 h in the final stage. Both the longer sintering and the more prolonged milling help with sintering. However, no apparent effect of the milling time can be seen on the density. The reason for the low values of the 6.0 h milling condition is unknown.

**Figure 7:**
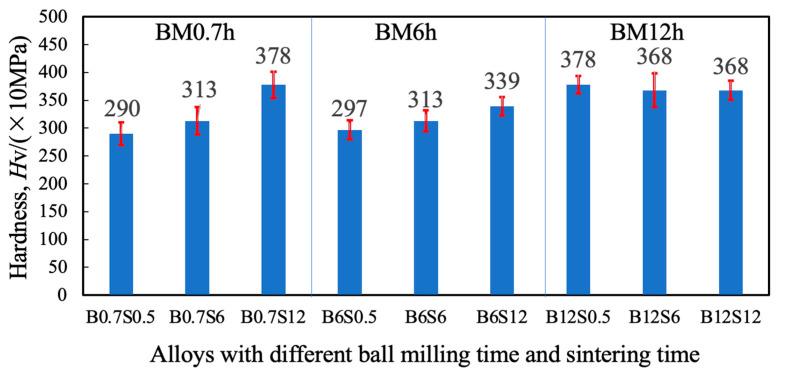
Vickers hardness of sintered CrMnFeCoNi alloys with different periods of milling and sintering.

Figure 7 shows the Vickers hardness obtained from the sintered specimens. The longer the milling or sintering, the higher hardness tended to be obtained. Compared to Figure 6, we know that hardness is related to densification. Otto reported a 660 MPa tensile intensity of Cantor alloy at room temperature [4]. Compared to this, the tensile strength can be derived from the hardness according to a conversion formula for SUS304 with the same fcc structure: 77.14 + 2.6396 Hv + 0.001 Hv^2^ [31]. By using the above formula, it can be found that the tensile intensity of B0.7S12 or B12S0.5 is the highest of 1218 MPa, about 1.5 times the above value.

Ball milling results in refining grains and changing lattice constants. In general, extended milling can help result in high-quality sintering. For the high-entropy alloy CrMnFeCoNi, the high-quality sintering corresponds to significant lattice distortion, producing high hardness through the solid solution strengthening mechanism [7]. In the case of 0.7 and 6.0 h milling, the effect of sintering time is apparent in the enhanced hardness. On the other hand, the effect became insensitive in the 12.0 h milled case; even the 0.5 h sintering achieved a high hardness. The slightly decreased strength with extended sintering in the case of 12.0 h milling might be relevant to the coarsening of crystalline size, which was detected from the XRD analysis (Section 3.2).

**Figure 8:**
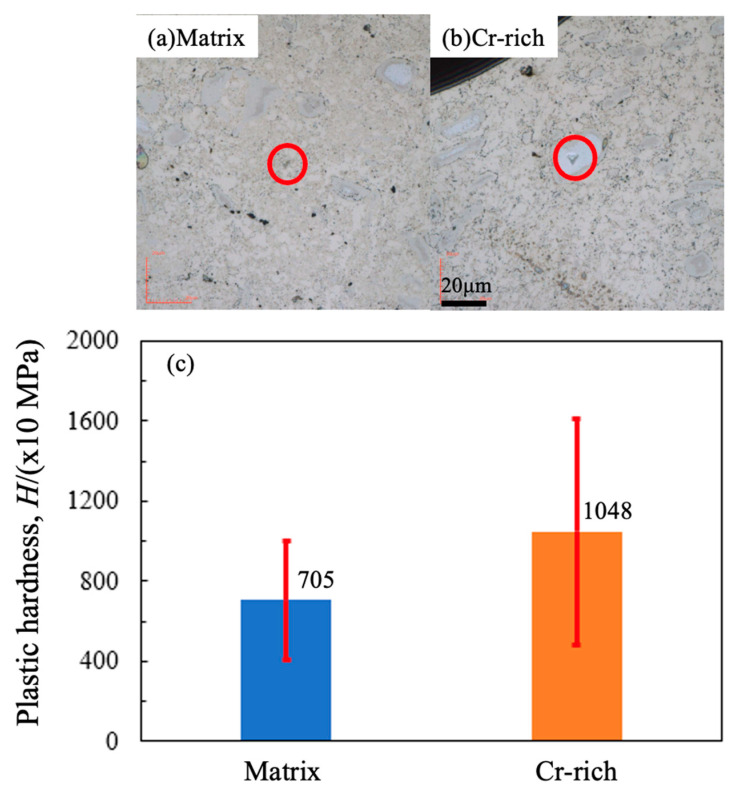
Plastic hardness of Cr-rich zone and matrix with a maximum force of 50 mN. Red circles show the dents on the matrix (**a**) and the Cr-rich zone (**b**). (**c**) shows the values of plastic hardness.

To clarify the effect of the Cr-rich zone on hardness, nanoindentation was applied to such zones with a maximum force of 50 mN using a Berkovich indenter. The results are shown in Figure 8. The hardness of the Cr-rich zone shows a significantly higher value than that of the matrix. Therefore, the dispersive Cr-rich zone might also have enhanced the hardness through the dispersion reinforcement effect.

In addition, the Vickers hardness of the sintered specimens of isolated metal was also measured. Values of 138 for Cr, 946 for Mn, 229 for Fe, 330 for Co, and 111 for Ni were obtained. Except for Mn, all are much lower than the sintered alloy. Therefore, CrMnFeCoNi showed a specific strength due to its high entropy characteristics [2].

### 3.4. Corrosion Resistance of Sintered Materials

**Figure 9:**
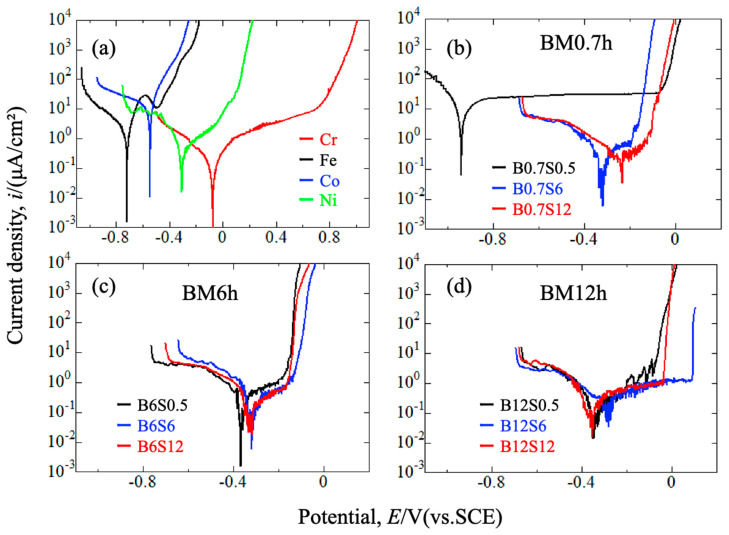
Polarization curves of sintered materials. (**a**) sintered Cr, Fe, Co, and Ni; CrMnFeCoNi alloys with 0.7 h (**b**), 6.0 h (**c**), and 12.0 h (**d**) milling.

Figure 9a shows the polarization curves of the sintered specimens of isolated Cr, Fe, Co, and Ni in a 3.5% NaCl solution. Mn was not measured due to its high brittleness in preparation. The stability can be obtained from the corrosion potential as Fe < Co < Ni < Cr, while the corrosion rate corresponds to the corrosion current density as Co > Fe > Ni > Cr. Passivity appeared on Fe, Ni, and Cr but not on Co. Generally, Cr shows the highest corrosion resistance due to forming a passive film. Figure 9b–d show the polarization curves of the sintered CrMnFeCoNi alloys. The lowest and the highest corrosion resistances were obtained on B0.7S0.5 and B12S6, respectively. Even the highest pitting potential of 95 mV vs. SCE is lower than that of conventional stainless steels such as SUS304 [32,33]. 

**Figure 10:**
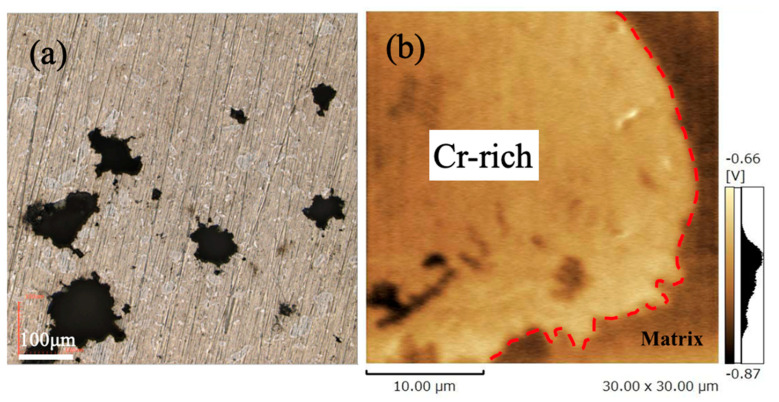
B12S0.5 surface after polarization (**a**) and the SKFM image of the matrix/Cr-rich zone (**b**).

Figure 10a shows an image of the corrosion surface of B12S0.5 after polarization. Irregular corrosion pits were observed in the same shape as the Cr-rich zones. The uncorroded Cr-rich zones appear white in this observation. Figure 10b shows a Kelvin potential image distribution around a Cr-rich zone and its surroundings for B12S0.5, measured by SKFM. The Cr-rich zone appeared to have higher potential than the matrix. Figure 10 explains the polarization result obtained in Figure 9b–d. The low and high pitting potentials are determined by the inhomogeneous degree of elements’ distribution in alloys, which change with milling and sintering conditions. Corrosion occurred around the Cr-rich zone due to the potential difference between the zone and the matrix since the Cr content in the matrix is much lower than the zone. Finally, the Cr-rich zone disappeared from the matrix. One reason for the lower pitting potential of all sintered alloys can be attributed to less Cr content in the matrix (Figure 4) and the potential difference (Figure 10b).

For the sintered alloys obtained with the 0.7 h milling process (Figure 9b), nobler corrosion potential and lower current density at the passive zone appeared for longer sintering. This corresponds to the enhanced entropy of the promoted atomic mixing and the formation of a condenser film due to the migration of Cr to the matrix. The above tendency also appeared on the sintered alloys with the 6.0 and 12.0 h milling processes. However, a monotonous increase in corrosion and pitting potentials with milling and sintering cannot be seen. This should be attributed to the progress of mixing and alloying in the 6.0 and 12.0 milling conditions, under which the effect of the sintering became somewhat undeterminable. As such, rather than applying extended milling and sintering, other essential parameters, such as the purity and the size of powders, the rotation rate and the ball/powder ratio in milling, and the temperature and the pressure in sintering, should be substantial. All these conditions aim to obtain a homogenous distribution of all elements, especially for Cr, which is the key to enhancing corrosion resistance by forming a condensed passive film.

According to the above measurements and analysis, CrMnFeCoNi alloy with high hardness was obtained by applying a suitable milling and sintering condition. Cr-rich zones appeared in the matrix, and had higher hardness than the matrix. This provides a possible dispersion reinforcement effect, except for the solid solution strength and the high entropy effects for CrMnFeCoNi. Mn, O, and C were contained in the zones; their solid solution or compound formation also helped to enhance the alloy’s strength. On the other hand, the inhomogeneous distribution of elements, especially the enriched Cr, is detrimental to the corrosion resistance in chloride solution. The lack of Cr content in the matrix cannot help form condensed passive films. The formation of Cr_23_C_6_ also promotes the inhomogeneous distribution of Cr. The content of O and C in the alloy should also degrade the corrosion resistance. Mn generally degrades the corrosion resistance [16,21]; therefore, the aggregated Mn in the Cr-rich zone should not be a helpful factor in enhancing the corrosion resistance. The homogeneously distribution of Cr to the matrix and the decrease in Mn on the surface are essential for further higher corrosion resistance.

## 4. Conclusions

A series of CrMnFeCoNi (Cantor) alloys were fabricated by ball milling and spark plasma sintering, with different milling and sintering times. Their crystal structure, density, hardness, and corrosion resistance were investigated, and their relationship was discussed. The following results were obtained.

1. Ball milling achieved a partial alloying of Cr, Mn, Fe, Co, and Ni at 300 rpm, with a higher alloying degree for a longer milling time.
2. Cr-rich zones, containing Mn, C, and O, formed in all sintered alloys, with inheritance from the ball-milled particles. Chromium carbide was confirmed in sintered alloys, whose amount was independent of milling time but decreased with the extended sintering.
3. Extended milling or long sintering can achieve high hardness and corrosion resistance for sintered alloys. The Cr-enriched zones, showing high hardness and potential, affect hardness and corrosion resistance.

## Figures and Tables

**Table 1:** Lattice constant and crystalline size of ball-milled powders (nm).

	BM0.7h	BM6h	BM12h
Lattice parameter	0.3525	0.3529	0.3532
Crystalline size	22	24	18

## Data Availability

Data are contained within the article.
